# Cortical mapping of mirror visual feedback training for unilateral upper extremity: A functional near‐infrared spectroscopy study

**DOI:** 10.1002/brb3.1489

**Published:** 2019-12-05

**Authors:** Zhongfei Bai, Kenneth N. K. Fong, Jiaqi Zhang, Zhishan Hu

**Affiliations:** ^1^ Department of Rehabilitation Sciences The Hong Kong Polytechnic University Kowloon Hong Kong SAR; ^2^ Department of Occupational Therapy Shanghai Yangzhi Rehabilitation Hospital (Shanghai Sunshine Rehabilitation Center) Shanghai China; ^3^ Department of Rehabilitation Sciences Tongji University School of Medicine Shanghai China; ^4^ Faculty of Health Sciences University of Macau Macau SAR

**Keywords:** deactivation, functional near‐infrared spectroscopy, mirror therapy, mirror visual feedback, precuneus, sensorimotor cortex, supplementary motor area

## Abstract

**Introduction:**

Mirror therapy has been shown to be effective in promoting hemiplegic arm recovery in patients with stroke or unilateral cerebral palsy. This study aimed to explore the cortical mapping associated with mirror therapy in a group of healthy adults by using functional near‐infrared spectroscopy.

**Methods:**

Fifteen right‐handed healthy adults were recruited by means of convenience sampling. A 2 × 2 factorial design was used: movement complexity with two levels—task‐based (T) and movement‐based (M), and visual direction with two levels—mirror visual feedback task (MT) and covered mirror with normal visual feedback task (NoT) as the control, constituting four conditions, namely TMT, MMT, TNoT, and MNoT. The regions of interest were the sensorimotor cortex (SMC), the supplementary motor area (SMA), the superior parietal cortex (SPL), and the precuneus in both the contralateral and ipsilateral hemispheres.

**Results:**

Our findings showed that in the ipsilateral hemisphere, MT induced a higher activation in the SMA and SPL than NoT. With regard to the activation of the ipsilateral SMC, only one channel was found showing superior effects of MT compared with NoT. In addition, MT can strengthen the functional connectivity between the SMC and SMA. In the contralateral hemisphere, both movement complexity and visual direction showed significant main effects in the SMC, while only movement complexity showed a significant main effect in the SMA and SPL. The precuneus of both sides was deactivated and showed no significant difference among the four conditions.

**Conclusions:**

Our experiment implies that the modest activation of ipsilateral SMC during MT is likely to be associated with the enhanced activity of ipsilateral SMA and that the precuneus may not be an essential component of the MT‐related neural network.

## Graphical abstract

Mirror therapy (MT) is a well‐studied neural rehabilitation technique for motor function recovery. However, the neural mechanisms and its neural substrates of MT have not been completely understood. In our study, we used functional near‐infrared spectroscopy to investigate the cortical mapping of MT over the following regions: sensorimotor cortex (SMC), supplementary motor area (SMA), superior parietal cortex (SPL), and precuneus. We found a slight activation in ipsilateral SMC induced by MT. Moreover, our finding showed that the SMA is essential for the activation of ipsilateral SMC. Our findings would like to argue that the precuneus is not an essential component of MT‐related neural network. We discussed why previous studies pointed out the precuneus was a special region for MT.

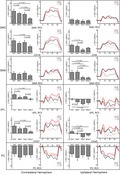

## 1 INTRODUCTION

Mirror visual feedback training (MT), also known as mirror therapy, is a well‐studied observation‐based motor learning strategy for the arm, in which participants are instructed to perform motor tasks with one hand (i.e., trained hand) while simultaneously viewing the mirror illusion of the moving hand from a mirror placed in their midsagittal plane, which has been proven to be useful in improving the motor functions of the hemiplegic arm in patients with stroke (Thieme et al., 2018; Toh & Fong, 2012). The mirror reflection of the trained hand makes it look as if the hand behind the mirror (i.e., the untrained hand) is moving, and this promotes the motor skills of the untrained hand, as has been reported in healthy adults (Nojima et al., 2012). A similar effect of mirror visual feedback of the unaffected arm has been found, and this can be considered as a therapeutic modality to train the hemiplegic (affected) arm of patients with stroke (Ramachandran, Rogers‐Ramachandran, & Cobb, 1995).

The effects of MT in motor recovery after stroke are always attributed to the increased excitability of the primary motor cortex (M1) ipsilateral to the moving arm induced by MT (i.e., ipsilesional M1 in patients with stroke; Pekna, Pekny, & Nilsson, 2012). Most previous studies using transcranial magnetic stimulation (TMS) found increased single‐pulse TMS‐induced motor evoked potentials (MEP) when receiving MT, compared with normal visual feedback (Fukumura, Sugawara, Tanabe, Ushiba, & Tomita, 2007; Garry, Loftus, & Summers, 2005; Kang, Ku, Kim, & Park, 2011). Other hypotheses were proposed to explain the mechanism of MT. First, the mirror neuron system, which discharges when observing other's actions and plays an important role in action understanding and imitation (Rizzolatti & Craighero, 2004), was found to be activated when observing mirror visual feedback (Matthys et al., 2009; Zhang, Fong, Welage, & Liu, 2018). Second, MT may recruit ipsilateral corticospinal projections from the unaffected hemisphere to the affected upper extremities. Third, attention toward the untrained side (i.e., affected arm in patients with stroke) can be increased due to the produced perceptual conflict between the visual feedback and the kinesthetic feedback. This latter hypothesis was supported by many studies showing increased activation in the cortical attention system, including the posterior cingulate cortex, insular cortex, superior parietal cortex (SPL), and the precuneus (Deconinck et al., 2015). Several studies, including one functional near‐infrared spectroscopy (fNIRS) study (Mehnert, Brunetti, Steinbrink, Niedeggen, & Dohle, 2013) and four functional magnetic resonance imaging (fMRI) studies (Dohle, Kleiser, Seitz, & Freund, 2004; Michielsen et al., 2011; Wang, Fritzsch, Bernarding, Holtze, et al., 2013; Wang, Fritzsch, Bernarding, Krause, et al., 2013), suggested that the precuneus might be engaged during MT. Previous studies about the precuneus revealed that it can become activated in various visuo‐spatial imagery tasks, such as motor imagery, mental rotation, and mental navigation (Cavanna & Trimble, 2006). However, as the precuneus is related to the default model network (DMN; Cunningham, Tomasi, & Volkow, 2017), its activity may decrease in response to attention‐demanding and non‐self‐referential tasks, in contrast to the resting state (Raichle, 2015). To perform movements with incongruent (i.e., mirrored) visual feedback is attention demanding; therefore, we expected to see a change in precuneus activity during MT. In addition, it was not consistently shown across fMRI studies that the activation of the supplementary motor area (SMA), which is related to the motor planning of self‐initiated movement and the learning of complicated motor skills, can be induced by MT (Matthys et al., 2009; Nachev, Kennard, & Husain, 2008; Shinoura et al., 2008).

In some clinical studies, researchers subdivided MT into two categories according to the complexity of the movements applied in the therapy: simple movement‐based MT (MMT), in which participants practice repetitive simple movements such as wrist flexion and extension, or finger flexion and extension, with their unaffected hand (Yavuzer et al., 2008); task‐based MT (TMT), in which participants perform specific task‐directed movements with their unaffected hand, such as squeezing sponges, placing pegs in holes, and flipping a card (Arya, Pandian, Kumar, & Puri, 2015). In terms of the difference between these two kinds of MT, the movements applied in TMT are more complex and attention demanding than the simple movements applied in MMT (Bai, Zhang, Zhang, Shu, & Niu, 2019). Recently, a study found increased cortical excitability of the M1 ipsilateral to the moving arm performing a precision grasp, which can be regarded as a task‐based movement (Jegatheeswaran, Vesia, Isayama, Gunraj, & Chen, 2018). Another study showed that the mirror condition with the target absent could not facilitate cortical excitability of the M1 ipsilateral to the moving arm, while the mirror condition with the target present could significantly increase it (Yarossi, Manuweera, Adamovich, & Tunik, 2017). However, the differential effects of these two kinds of MT are still unclear because of the limited amount of studies.

Functional near‐infrared spectroscopy is a noninvasive neuroimaging technique that measures cortical activity by detecting the hemodynamic responses associated with neural activity. The spatial resolution of fNIRS is intermediate between fMRI and electroencephalography. Compared with fMRI, fNIRS has the advantage of portability, which makes it a promising tool in investigating some neurobehavioral questions (Cui, Bray, Bryant, Glover, & Reiss, 2011). In the present study, the aims were twofold. First, we aimed to investigate the cortical activation associated with MT, particularly in the sensorimotor cortex (SMC), SMA, SPL, and precuneus, using fNIRS. Second, we expected to explore the effect of movement complexity during MT by comparing two categories of MT (i.e., TMT and MMT).

## 2 METHODS

### 2.1 Participants

Since the present study was not gender‐related, and due to the advantages of uniformity of head size and shorter hair, 15 male volunteers were enrolled in this study via convenience sampling. All the participants were aged between 22 and 31 years (mean = 26.93, *SE* = 0.71) and right‐handed according to self‐report. The study was conducted following the ethical principles regarding human experiments (Helsinki Declaration; Christie, 2000). Written informed consent was obtained from all participants prior to data collection. The study was approved by the Human Research Ethics Committee of the Hong Kong Polytechnic University (Reference Number: HSEARS20181221001).

### 2.2 Experimental design and procedure

**Figure 1:**
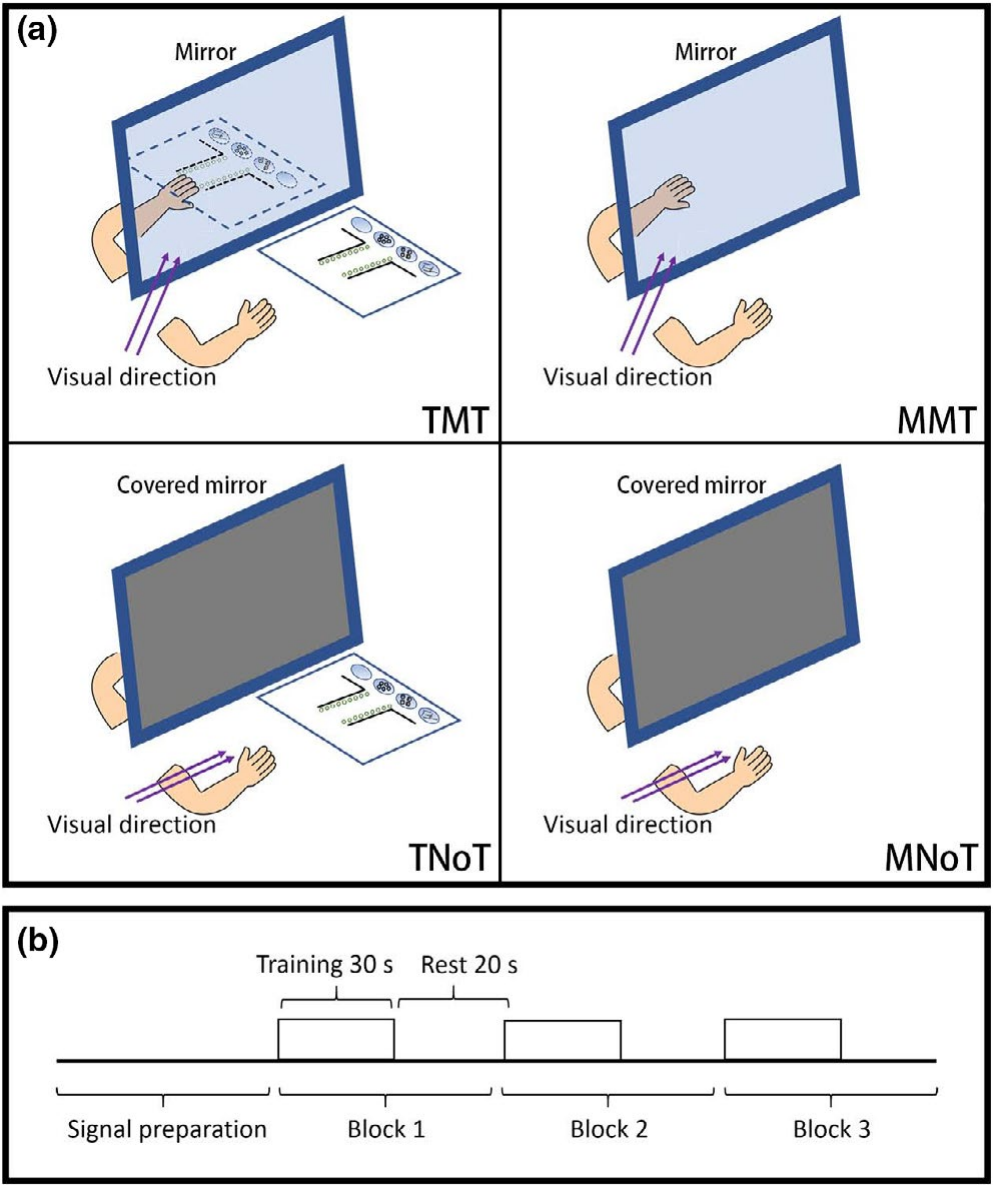
Experimental design. (a) Experimental setup. (b) Experimental design. MMT, movement‐based mirror therapy; MNoT, movement‐based with normal visual feedback; TMT, task‐based mirror therapy; TNoT, task‐based with normal visual feedback

The present study had a 2 × 2 factorial design. The first factor was movement complexity with two levels: task‐based (T) and movement‐based (M). The second factor was visual direction with two levels: mirror visual feedback task (MT) and covered mirror with normal visual feedback task (NoT) as the control. Each participant completed the four experimental conditions depicted in Figure 1a: TMT, MMT, task‐based with normal visual feedback (TNoT), and movement‐based with normal visual feedback (MNoT). The experimental setup of the four conditions was also shown in a video available online (see Video S1).

Participants were seated comfortably on an adjustable chair in front of a table in a room that was quiet except for the consistent noise made by an fNIRS machine. A computer was placed on the table about 70 cm away from the participants. For the setups of the MT paradigm, we used a physical mirror (43 cm × 40 cm) to simulate a real clinical MT practice. The motor task in both the TMT and TNoT conditions was modified from the assembly task of the Purdue Pegboard Test. Originally, this task was used to assess bilateral hand dexterity by counting how many assemblies are completed in 60 s (Yeudall, Fromm, Reddon, & Stefanyk, 1986). In our experiment, participants were instructed to perform the assembly tasks by picking up small objects (pins, washers, and collars) with their right hand only. In the TMT condition, participants were instructed to perform the assembly task as fast as they could while simultaneously looking at the mirror reflection of their trained hand. During the TMT, the Purdue Pegboard was positioned 30 cm away from the mirror to avoid any glimpses at the real Purdue Pegboard. In the TNoT condition, a covered mirror (i.e., a nontransparent board) was used and participants were instructed to complete the task while looking at their moving hand directly.

The setups in the MMT and MNoT conditions were similar to those in the TMT and TNoT conditions, but the Purdue Pegboard was removed. Participants performed the same picking‐up movements without any objects. A previous study reported that the normative data of this assembly task in adults were around 30 repetitions in 60 s (Yeudall et al., 1986). Therefore, to match the quantity of repetitions in the task‐based conditions, participants were required to perform the MMT and MNoT at a pace of 0.5 Hz. To familiarize themselves with this pace, participants practiced the pace alone with a metronome before the experiment. In the MMT condition, participants always looked at the mirror reflection while performing the motor task. In the MNoT condition, a covered mirror replaced the real mirror and participants looked at their moving hand directly.

To allow participants to familiarize themselves with these four conditions, each participant was allowed a 5‐min practice session. Participants were allowed a 5‐min break between each condition. The sequence of conditions for each participant was randomly allocated using the E‐Prime software (version 2; Psychology Software Tools, Inc.) to avoid any bias from the sequence. As shown in Figure 1b, a block design paradigm was employed for the acquisition of fNIRS data. Each condition was started once the fNIRS signal was stable, and each condition consisted of three blocks. Each block consisted of a 30‐s task period and a 20‐s rest period. During the rest period, participants focused on a white cross on a black background presented on a monitor and were asked to remain motionless. The start and end signs of blocks were given by a pure tone (“ding”) using the E‐Prime software. All participants completed the four conditions in 1 hr.

### 2.3 fNIRS data acquisition

**Figure 2:**
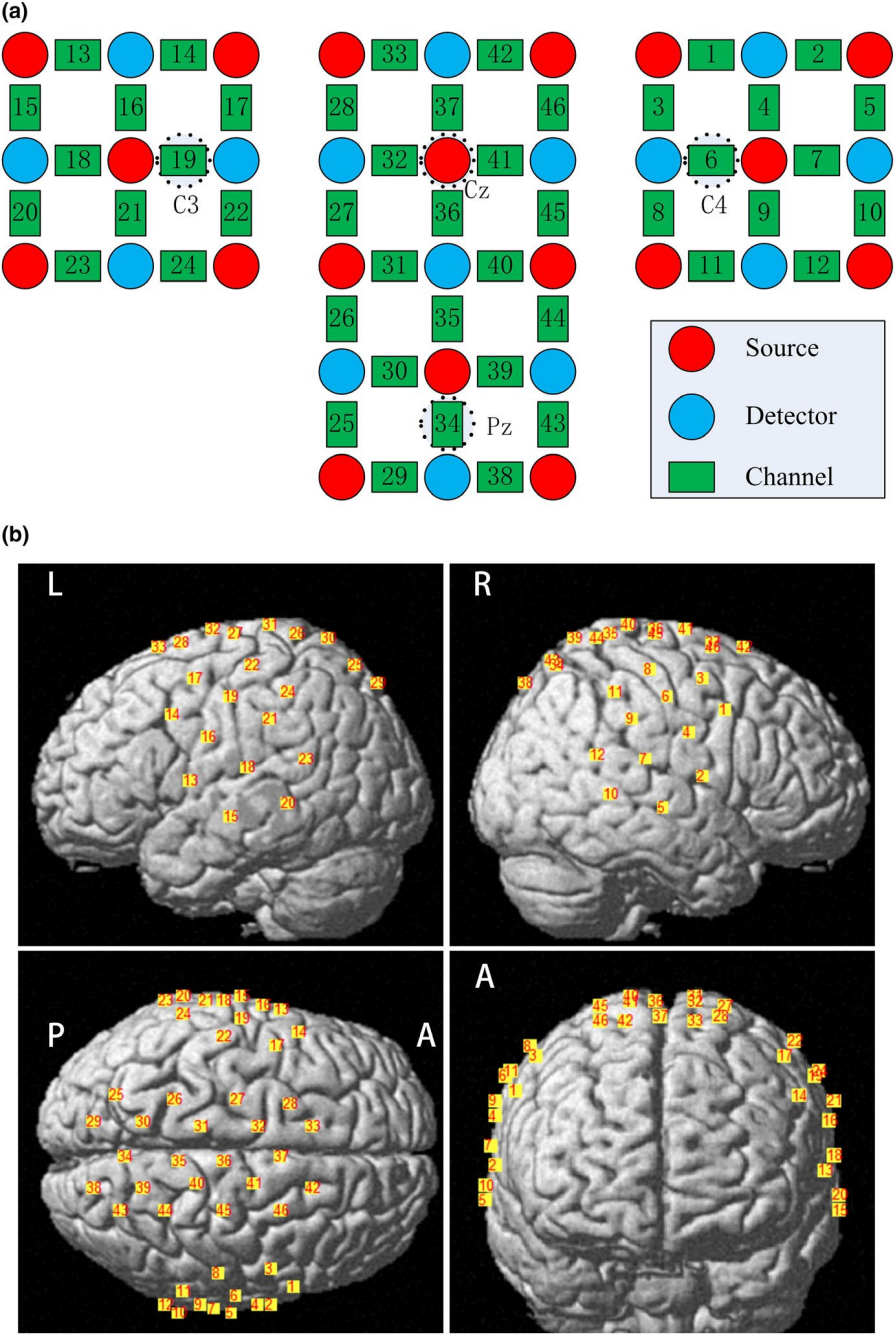
Arrangement of fNIRS channels. (a) Two 3 × 3 and one 3 × 5 optode probe sets. (b) The locations of channels in the MNI standard brain template

In this study, the concentration changes of oxyhemoglobin (ΔHbO) and deoxyhemoglobin (Hb) at the cortex were measured by a continuous‐wave optical system (ETG‐4000, Hitachi Medical Co.). The sources of this system generate two wavelengths of near‐infrared light at 690 and 830 nm, and the sampling rate is fixed at 10 Hz. We used three probe sets consisting of two 3 × 3 and one 3 × 5 optode probe sets (Figure 2a). A total of 18 light sources and 15 detectors with an inter‐optode distance of 3 cm constituted 46 channels (CH) to allow the measurement of the central and posterior cortices. Specifically, the source at the second row and middle column was positioned at the Cz in accordance with the international 10–20 system, while CH19 and CH6 were positioned at C3 and C4, respectively. Presumably, CH34 was located at the Pz where the projection of the precuneus was. To localize the coordinates of each channel in the MNI standard brain template (Lancaster et al., 2000), a 3D digitizer (PATRIOT, Polhemus) was used, and the coordinates were further imported to the NIRS_SPM toolbox for spatial registration (available at: https://www.nitrc.org/projects/nirs_spm/; Ye, Tak, Jang, Jung, & Jang, 2009). Figure 2b shows the location of each channel in the MNI standard brain template. We defined regions of interest (ROIs) for subsequent analysis with references to the MNI coordinates and the 10–20 system. CH17, CH16, and CH19 served as anatomic markers for the left SMC; CH3, CH4, and CH6 for the right SMC; CH28 and CH33 for the left SMA; CH46 and CH42 for the right SMA; CH26 and CH27 for the left SPL; CH44 and CH45 for the right SPL; CH25, CH29, and CH30 for the left precuneus; and CH43, CH38, and CH39 for the right precuneus.

### 2.4 Preprocessing of fNIRS data

In this study, we adopted HbO signals as the indicator of hemodynamic response since HbO is more sensitive to regional cerebral blood flow than Hb (Hoshi, Kobayashi, & Tamura, 2001; Strangman, Culver, Thompson, & Boas, 2002). The HomER2 toolbox in Matlab 2014a (The MathWorks Inc.) was used for offline data preprocessing (Huppert, Diamond, Franceschini, & Boas, 2009). After the raw intensity data were converted to optical density changes, the spline interpolation algorithm was used to correct motion artifacts caused by head movement during data acquisition. Then, a bandpass filter between 0.01 and 0.2 Hz was applied to remove the effect of physiological noises and drifts. Finally, the optical density was converted to ΔHbO on the basis of the modified Beer‐Lambert law. We cut a temporal window from −5 to 40 s relative to the onset of blocks (*t* = 0 s) for averaging. Since around five seconds is needed for HbO to increase from baseline to a stable concentration change, the time course of HbO from 5 to 30 s was averaged to obtain the mean ΔHbO induced by the conditions (Aarabi, Osharina, & Wallois, 2017).

### 2.5 Statistical analysis

The average of multiple channels serving as one ROI on ΔHbO was calculated for ROI‐level statistical analysis. We analyzed each individual channel in ROIs in case of an insignificant result in the ROI‐level comparison. Functional connectivity (FC) among channels was also analyzed. The node was defined as the channels, and the edge was defined as the Pearson correlation coefficient between each pair of channels. FC was visualized using the BrainNet Viewer toolbox (Xia, Wang, & He, 2013).

Data analysis was conducted using SPSS (version 23.0; SPSS Inc.). The level of significance was set at *p* < .05 (two‐tailed). In this study, the cortical activation was subjected to a 2 × 2 repeated measures analysis of variance (ANOVA), with two main effects (i.e., movement complexity and visual direction) and one interaction effect (movement complexity × visual direction). Post hoc analysis was performed by using paired *t* test between conditions (TMT vs. MMT, TMT vs. TNoT, MMT vs. MNoT, TNoT vs. MNoT), and Bonferroni correction was applied to avoid the inflation of type I error (corrected alpha threshold = 0.05/4). To compare the FC between conditions, the Pearson correlation coefficient between channels was transformed into a *z*‐score with the Fisher transformation. Then, the *z*‐scores were compared with the *Z* statistic to determine the difference in FC between conditions.

## 3 RESULTS

**Figure 3:**
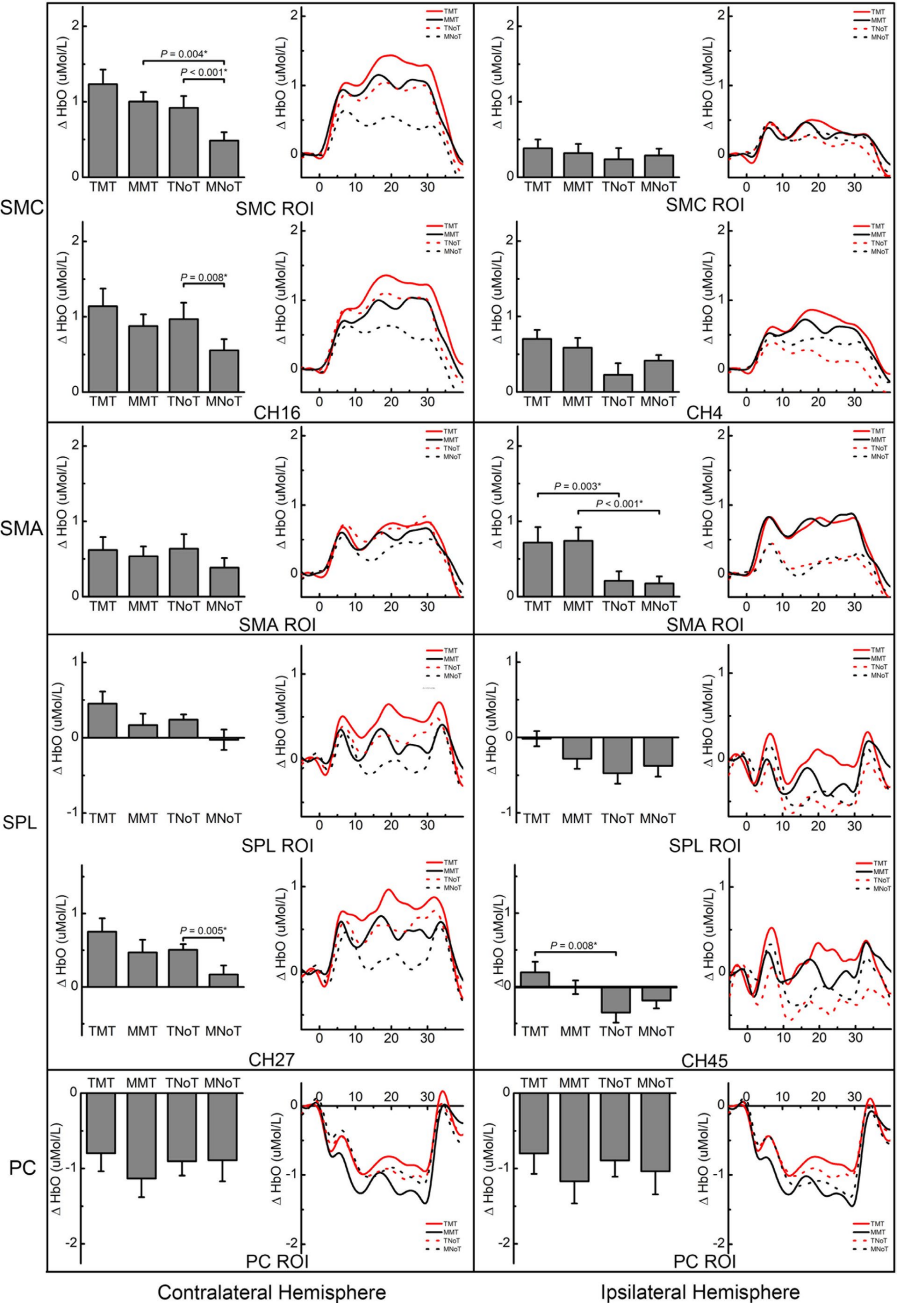
Post hoc analysis and baseline‐corrected time course curves. The bar charts show the post hoc paired *t* test analysis, and the line charts show the baseline‐corrected time course curves between −5 and 40 s relative to the onset of tasks. *: Post hoc analysis which passed the Bonferroni correction at *p* ≤ .013 (0.05/4; 4 = number of comparisons); CH, channel; MMT, movement‐based mirror therapy; MNoT, movement‐based with normal visual feedback; PC, precuneus; ROI, region of interest‐based analysis; SMA, supplementary motor area; SMC, sensorimotor cortex; TMT, task‐based mirror therapy; TNoT, task‐based with normal visual feedback [Correction added on 20 December 2019, after first online publication: Figure 3 has been updated and *p* values in caption have been corrected.]

Table 1 shows the results of a two‐way repeated measures ANOVA in terms of the main effects of visual direction and movement complexity and their interaction effect on ΔHbO in the SMC, SMA, SPL, and precuneus. Post hoc analysis and the baseline‐corrected time course curve are shown in Figure 3.

**Table 1:** Two‐way ANOVA analysis on HbO change in the M1, SMA, SPL, and PC

Cortical region			TMT	MMT	TNoT	MNoT	Main effects				Interaction effect	
							Visual direction		Movement complexity			
							*F* value	*p* value	*F* value	*p* value	*F* value	*p* value
SMC	Contra	ROI	1.23 ± 0.19	1.00 ± 0.13	0.92 ± 0.16	0.49 ± 0.11	7.54	.016*	16.44	.001*	3.62	.078
		CH16	1.14 ± 0.23	0.88 ± 0.15	0.97 ± 0.22	0.55 ± 0.15	1.13	.306	7.72	.015*	0.95	.347
		CH17	1.06 ± 0.20	0.90 ± 0.13	0.81 ± 0.17	0.43 ± 0.12	6.64	.022*	6.66	.023*	2.72	.121
		CH19	1.49 ± 0.23	1.23 ± 0.16	0.98 ± 0.14	0.47 ± 0.11	15.15	.002*	15.16	.002*	3.10	.100
	Ipsi	ROI	0.38 ± 0.12	0.32 ± 0.12	0.24 ± 0.15	0.29 ± 0.09	0.40	.538	0.01	.947	1.33	.268
		CH4	0.70 ± 0.12	0.59 ± 0.13	0.23 ± 0.15	0.41 ± 0.07	8.16	.013*	0.14	.712	2.36	.147
		CH3	0.12 ± 0.17	0.05 ± 0.20	0.20 ± 0.18	0.23 ± 0.13	0.37	.552	0.05	.823	0.52	.482
		CH6	0.33 ± 0.17	0.33 ± 0.16	0.28 ± 0.15	0.22 ± 0.11	0.17	.686	0.17	.690	0.57	.463
SMA	Contra	ROI	0.62 ± 0.17	0.53 ± 0.13	0.64 ± 0.19	0.38 ± 0.13	0.31	.586	3.72	.074	1.36	.263
		CH28	0.66 ± 0.17	0.50 ± 0.12	0.76 ± 0.20	0.40 ± 0.12	<0.01	.996	6.43	.024*	1.74	.208
		CH33	0.58 ± 0.20	0.57 ± 0.19	0.51 ± 0.22	0.37 ± 0.15	0.62	.445	0.69	.421	0.51	.489
	Ipsi	ROI	0.72 ± 0.21	0.74 ± 0.18	0.21 ± 0.13	0.18 ± 0.09	20.97	<.001*	<0.01	.957	0.37	.552
		CH46	0.82 ± 0.20	0.80 ± 0.19	0.11 ± 0.10	0.13 ± 0.10	29.91	<.001*	<0.01	.984	0.10	.762
		CH42	0.62 ± 0.22	0.69 ± 0.18	0.31 ± 0.17	0.22 ± 0.11	8.17	.013*	0.01	.909	1.96	.183
SPL	Contra	ROI	0.45 ± 0.16	0.17 ± 0.15	0.24 ± 0.07	−0.03 ± 0.14	2.81	.116	11.69	.004*	0.02	.903
		CH26	0.16 ± 0.18	−0.13 ± 0.19	−0.03 ± 0.09	−0.22 ± 0.18	1.32	.269	5.33	.037*	0.31	.589
		CH27	0.75 ± 0.18	0.47 ± 0.17	0.51 ± 0.08	0.17 ± 0.12	2.72	.121	14.46	.002*	0.14	.715
	Ipsi	ROI	−0.02 ± 0.10	−0.28 ± 0.13	−0.48 ± 0.14	−0.38 ± 0.14	5.37	.036*	0.65	.434	5.07	.041*
		CH44	−0.23 ± 0.16	−0.56 ± 0.21	−0.60 ± 0.17	−0.56 ± 0.20	1.49	.242	1.41	.255	4.38	.055
		CH45	0.19 ± 0.15	−0.01 ± 0.09	−0.35 ± 0.14	−0.19 ± 0.11	10.80	.005*	0.03	.866	3.91	.068
PC	Contra	ROI	−0.80 ± 0.24	−1.13 ± 0.25	−0.90 ± 0.19	−0.89 ± 0.28	0.19	.670	0.62	.443	2.84	.114
		CH25	−0.88 ± 0.30	−1.28 ± 0.27	−0.88 ± 0.24	−0.92 ± 0.32	0.86	.370	1.02	.331	2.13	.167
		CH29	−1.23 ± 0.28	−1.64 ± 0.33	−1.35 ± 0.28	−1.26 ± 0.34	0.58	.459	0.25	.626	4.44	.054
		CH30	−0.27 ± 0.21	−0.48 ± 0.23	−0.48 ± 0.10	−0.49 ± 0.20	0.47	.505	1.12	.307	1.28	.277
	Ipsi	ROI	−0.80 ± 0.27	−1.17 ± 0.29	−0.89 ± 0.22	−1.04 ± 0.31	0.01	.922	1.58	.229	0.88	.229
		CH43	−0.93 ± 0.29	−1.37 ± 0.32	−1.11 ± 0.33	−1.24 ± 0.38	0.01	.915	1.43	.252	0.92	.354
		CH38	−1.09 ± 0.36	−1.48 ± 0.38	−1.06 ± 0.24	−1.33 ± 0.37	0.20	.661	1.25	.282	0.14	.712
		CH39	−0.38 ± 0.20	−0.65 ± 0.25	−0.52 ± 0.16	−0.54 ± 0.20	<0.01	.962	1.37	.262	2.41	.143

Note

Values represent mean ± *SEM*, unit: μMol/L;

* *p* ≤ .05.

Abbreviations: CH, channel; Contra, contralateral hemisphere; HbO, oxyhemoglobin; Ipsi, ipsilateral hemisphere; MMT, movement‐based mirror therapy; MNoT, movement‐based with normal visual feedback; PC, precuneus; ROI, region of interest‐based analysis; SMA, supplementary motor area; SMC, sensorimotor cortex; TMT, task‐based mirror therapy; TNoT, task‐based with normal visual feedback.

### 3.1 Cortical activation of the SMC

The two‐way ANOVA showed significant main effects of visual direction (*F* = 7.54, *p* = .016) and movement complexity (*F* = 16.44, *p* = .001) on ΔHbO in the contralateral SMC, while the interaction effect was insignificant (*F* = 3.62, *p* = .078). For the individual channels constituting the contralateral SMC, a significant main effect of visual direction in CH17 (*F* = 6.64, *p* = .022) and CH19 (*F* = 15.15, *p* = .002) and a significant main effect of movement complexity in CH16 (*F* = 7.72, *p* = .015), CH17 (*F* = 6.66, *p* = .023), and CH19 (*F* = 15.16, *p* = .002) were found. However, no significant interaction effects were noted in CH16 (*F* = 0.95, *p* = .347), CH17 (*F* = 2.72, *p* = .121), and CH19 (*F* = 3.10, *p* = .100).

As regards the activation of the ipsilateral SMC, there was no significant main effect of either visual direction (*F* = 0.40, *p* = .538) or movement complexity (*F* = 0.01, *p* = .947) and also no significant interaction effect (*F* = 1.33, *p* = .268) on ΔHbO. However, there was a significant main effect of visual direction (*F* = 8.16, *p* = .013) on ΔHbO in CH4 only, while the main effect of movement complexity (*F* = 0.14, *p* = .712) and the interaction effect (*F* = 2.36, *p* = .147) were insignificant. Post hoc analysis showed that TMT was superior to TNoT (*t* = 2.84, *p* = .013), but the comparison did not pass the Bonferroni correction. The comparison between TMT and MMT was not significant (*t* = 1.24, *p* = .237).

### 3.2 Cortical activation of the SMA

The two‐way ANOVA showed that the main effects of visual direction (*F* = 0.31, *p* = .586) and movement complexity (*F* = 3.74, *p* = .074) and the interaction effect (*F* = 1.36, *p* = .263) were not significant on ΔHbO in the contralateral SMA. However, a significant main effect of movement complexity was found in CH28 (*F* = 6.43, *p* = .024), indicating that task‐based task can elicit a higher level of activation in the contralateral SMA than movement‐based task.

On the contrary, a significant main effect of visual direction was found on ΔHbO in the ipsilateral SMA (*F* = 20.97, *p* < .001), indicating the superior effect of mirror visual feedback in activating the ipsilateral SMA. We did not find a significant main effect of movement complexity (*F* < 0.01, *p* = .957) and the interaction effect (*F* = 0.37, *p* = .552) on ΔHbO in the ipsilateral SMA. Post hoc analysis did not show any significant difference between TMT and MMT (*t* = 0.833, *p* = .419). Channel‐level analysis in CH46 and CH42 showed similar results to the analysis based on ROI.

### 3.3 Cortical activation of the SPL

The two‐way ANOVA showed that the main effect of movement complexity on ΔHbO in the contralateral SPL was significant (*F* = 11.69, *p* = .004), while the main effect of visual effect (*F* = 2.81,* p* = .116) and the interaction effect (*F* = 0.02, *p* = .903) were not significant, indicating the superior effect of task‐based task in activating the contralateral SPL. Channel‐level analysis in CH26 and CH27 showed similar results to the analysis based on ROI.

In the ipsilateral SPL, ΔHbO tended to have deactivation instead of activation. We found a significant main effect of visual direction (*F* = 5.37, *p* = .036) and a significant interaction effect (*F* = 5.07,* p* = .041) on ΔHbO in the ipsilateral SPL, indicating that mirror visual feedback induced less deactivation than the covered mirror. Post hoc analysis showed that TMT tended to induce less deactivation than MMT (*t* = 2.66, *p* = .019). Furthermore, in CH45, the main effect of visual direction was more robust (*F* = 10.80, *p* = .005), and TMT tended to revise the deactivation to activation.

### 3.4 Cortical activation of the precuneus

We found deactivation in the precuneus instead of activation in the four conditions. The two‐way ANOVA showed no significant main or interaction effects on ΔHbO in the contralateral and ipsilateral precuneus areas. Similarly, we found no significant main or interaction effects in any channel‐based analysis.

### 3.5 Functional connectivity

**Figure 4:**
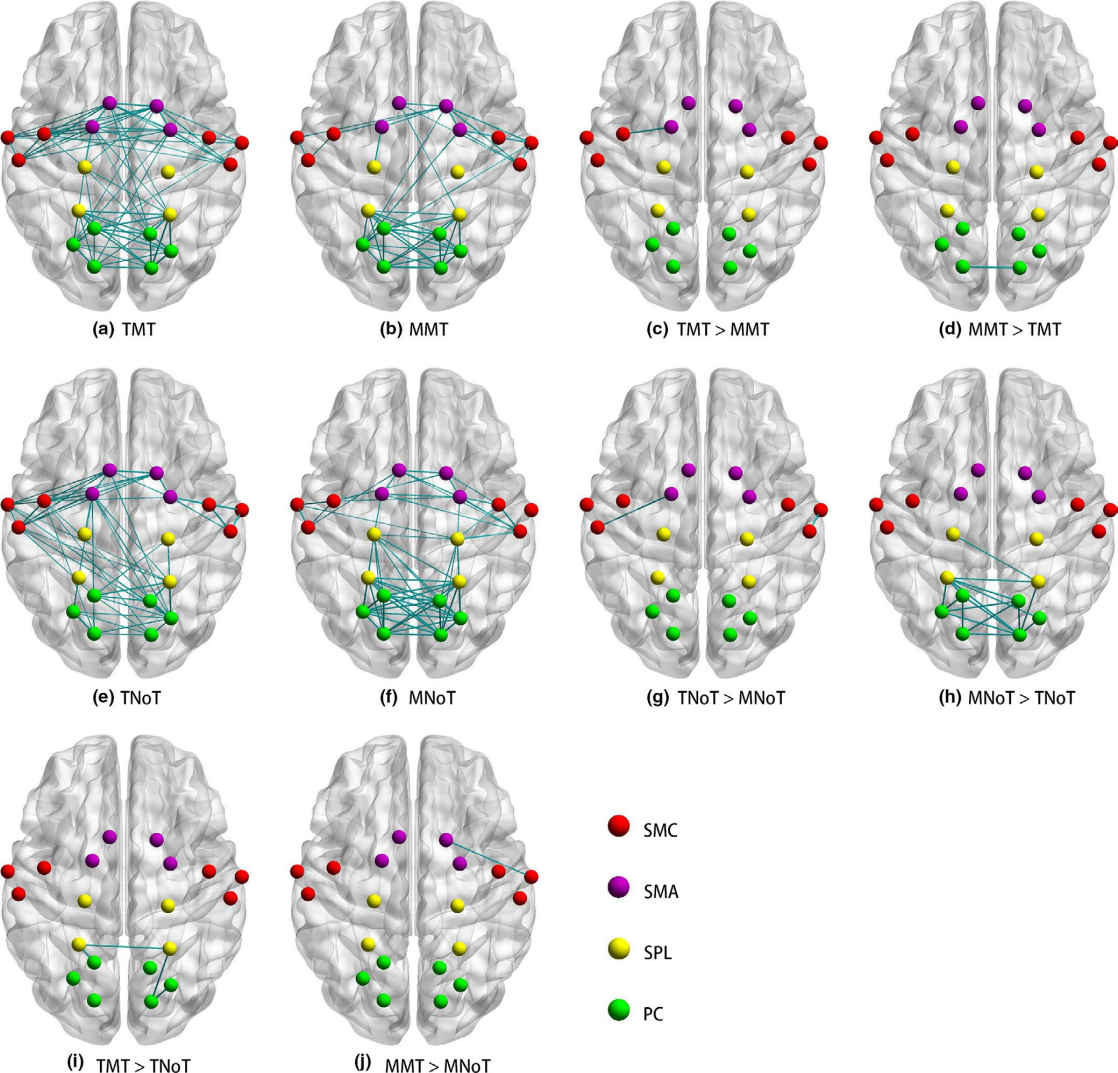
The differences of functional connectivity among conditions. a, b, e, and f show the significant functional connectivity of task‐based mirror therapy (TMT), movement‐based mirror therapy (MMT), task‐based with normal visual feedback (TNoT), and movement‐based with normal visual feedback (MNoT), respectively. c, d, g, h, i, and j show comparisons between conditions. “>” means functional connectivity whose left condition is stronger than right condition. PC, precuneus; SMA, supplementary motor area; SMC, sensorimotor cortex

With regard to the effect of MT, MMT produced significantly increased FC between the ipsilateral SMA and the ipsilateral SMC compared with MNoT (*Z* = 2.30, *p* = .021; Figure 4j). Although TMT likewise showed superior benefits compared with TNoT, the comparison was not significant, as shown in Figure 4i. As regards the effect of task‐based task, TMT produced significantly stronger FC between the contralateral SMA and the contralateral SMC than MMT (*Z* = 2.51, *p* = .012; Figure 4c), and TNoT likewise produced significantly stronger FC between the contralateral SMA and the contralateral SMC than MNoT (*Z* = 2.42, *p* = .016; Figure 4g).

## 4 DISCUSSION

In the present study, we investigated the cortical activation of MT with motor tasks of different complexities in a group of healthy adults using fNIRS. Four ROIs, namely the SMC, SMA, SPL, and precuneus in both the contralateral and ipsilateral hemispheres, were predefined and then explored. The concentration change of HbO was used as the indicator of cortical activity in the four conditions: TMT, MMT, TNoT and MNoT. In addition, the differences of FC among conditions were calculated.

### 4.1 Cortical activation of the SMC

We found that mirror visual feedback induced higher activation in the ipsilateral SMC compared with normal visual feedback. However, we also realized that the main effect of visual direction was shown only in one channel, and the post hoc analysis did not pass the Bonferroni correction. In this present fNIRS study, we cannot differentiate the M1 from the primary sensory cortex because the spatial resolution of fNIRS is not high enough (Cui et al., 2011). In studies employing fMRI, which compared mirror and normal visual feedbacks, most did not find significant activation in the M1 ipsilateral to the moving hand during MT in either healthy (Hamzei et al., 2012; Matthys et al., 2009; Wang, Fritzsch, Bernarding, Holtze, et al., 2013) or stroke (Michielsen et al., 2011; Saleh, Adamovich, & Tunik, 2014; Shinoura et al., 2008) populations. In a recent fNIRS study, 3D T1‐weighted MRI was used for the anatomical location of NIRS channels (Inagaki et al., 2019), and researchers found that mirror visual feedback applied in unilateral hand movement could induce higher activation in the postcentral gyrus but not in the precentral gyrus. Moreover, previous fMRI studies also confirmed that MT is able to modulate the activation of somatosensory areas (Fritzsch et al., 2014; Saleh et al., 2014). Therefore, the activation in the ipsilateral SMC that we found in the present study might result from the activation of somatosensory areas rather than motor areas. On the other hand, many TMS studies have indicated that the corticospinal excitability of the ipsilateral M1 increases, as reflected by increased MEP, in both healthy and stroke participants during MT (Funase, Tabira, Higashi, Liang, & Kasai, 2007; Garry et al., 2005; Kang et al., 2011). Two studies did not find the increment of MEP induced by MT, but they reported a decrement of short‐interval intracortical inhibition, which can be considered as another indicator associated with activation within the motor cortex (Jegatheeswaran et al., 2018; Kumru et al., 2016). Taken together, most TMS studies support the activation of M1 caused by MT, while hemodynamic response‐based neuroimaging studies, such as fMRI and fNIRS, fail to show the activation of M1. A previous study investigating the relationship between neuronal activity and corresponding hemodynamic response showed that synaptic activity has to reach a threshold before increasing in local flow proportionately (Sheth et al., 2004). Therefore, we speculate that MT is likely to modulate the neural activity of the ipsilateral M1, but the effect might be too weak to be detected by fMRI and fNIRS.

We found that MT also significantly activated the contralateral SMC, which is consistent with a previous fMRI study (Fritzsch et al., 2014). So far, some researchers have proposed that recruiting the corticospinal tract from the contralesional hemisphere may facilitate the recovery of affected arms (Jankowska & Edgley, 2006). Therefore, the recruitment of the contralesional corticospinal tract might be a potential neural correlate underlying the effect of MT in patients with stroke which deserves more systematic investigation.

### 4.2 Cortical activation on other areas

Several previous neuroimaging studies showed inconsistent findings in regard to the activation of the SMA under the MT paradigm (Fritzsch et al., 2014; Michielsen et al., 2011; Saleh et al., 2014; Shinoura et al., 2008). In the present study, we found that MT induced significantly higher activation in the ipsilateral SMA, in contrast to normal visual feedback, which is consistent with a recent fNIRS study (Inagaki et al., 2019); however, the main effect of movement complexity was insignificant. On the other hand, we found that task‐based task tended to induce higher activation than movement‐based task in the contralateral SMA, while the main effect of visual direction was insignificant. Furthermore, we found that the FC between the ipsilateral SMA and the ipsilateral SMC was stronger in the MT conditions than in the normal visual feedback conditions. The SMA is important for the initiation of movement, the orientation of the eyes and head, and the planning of bimanual and sequential movements (Lundy‐Ekman, 2007). We considered that MT might be able to recruit the SMA via two potential mechanisms: first, the mirror neuron system and, second, the cortical‐basal ganglia circuit. A previous clinical study found that multiple sessions of action observation training could significantly enhance the activity of the SMA in patients with stroke, which indicated that the SMA is responsive to action observation (Ertelt et al., 2007). According to a meta‐analysis (Molenberghs, Cunnington, & Mattingley, 2009), the SMA has been shown to have the mirror neuron‐like property which is involved during action observation and imitation. The activated mirror neuron system is assumed to be an essential neural network for observation‐based motor learning (Cook, Bird, Catmur, Press, & Heyes, 2014). MT may have similar effects as action observation on recruiting the SMA. Second, MT is likely to recruit the SMA to facilitate visual‐motor integration by stimulating the basal cortical–basal ganglia circuit. This circuit starts with the projection from the association areas in the frontal and parietal lobes to the SMA and pre‐SMA via the basal ganglia (Freeze, Kravitz, Hammack, Berke, & Kreitzer, 2013). Anatomically, the pre‐SMA makes a direct contribution to the corticospinal tract and the SMA projects to the M1 (Nachev et al., 2008), and the high‐frequency excitatory TMS of the SMA can enhance the excitability of the M1 (Lu, Arai, Tsai, & Ziemann, 2012).

The SPL is a part of the dorsal frontoparietal attention network dominating the voluntary allocation of attention, and it shows higher activation when attention is selective to the contralateral visual field (Corbetta & Shulman, 2002). In our study, we found that task‐based task produced higher activation in the contralateral SPL than movement‐based task, which was reasonable due to the high level of complexity of motor tasks. On the other hand, we found that the activity of ipsilateral SPL was deactivated in normal visual feedback conditions which was in line with a previous fMRI study (Marchand et al., 2007) that mirror visual feedback tended to revise the state from deactivation to activation in ipsilateral SPL. Moreover, TMT could produce higher neural activity than MMT in the ipsilateral SPL. However, the activation in the ipsilateral SMC as well as the FC between the SPL and the SMC was not significantly different (Figure 4c,d). Furthermore, compared with normal visual feedback, MT did not produce increased FC between the SPL and the SMC. Taken together, although we found that MT induced superior effects in the activation of the SPL, the SPL seemed irrelevant to the activation of the SMC induced by MT. Therefore, the potential relationship between the increased activation in the SPL and the increased activation in the SMC in MT awaits further exploration.

We found deactivation in the precuneus instead of activation, and the results did not show any significant difference among the conditions. For fMRI studies, the blood oxygenation level‐dependent (BOLD) signal contrast is essential to interpret the signals as activation or deactivation (Frankenstein et al., 2003). In previous fMRI studies, several experiments showed that compared with normal visual feedback conditions, MT can activate the precuneus (Dohle et al., 2004; Michielsen et al., 2011; Wang, Fritzsch, Bernarding, Holtze, et al., 2013; Wang, Fritzsch, Bernarding, Krause, et al., 2013). Nevertheless, most studies did not perform the BOLD contrasts between conditions and baselines, but rather only the contrasts among conditions (Dohle et al., 2004; Michielsen et al., 2011; Saleh et al., 2014). Wang, Fritzsch, Bernarding, Holtze, et al. (2013) provided the beta values with 90% confidence intervals of each condition which can be used for the contrast. Their results certainly showed that the beta value of the ipsilateral precuneus relative to the moving hand during MT was higher than that in normal visual feedback conditions. However, first, we realized that the beta values in normal visual feedback conditions were negative, which meant that the precuneus showed deactivation instead of activation in the normal visual feedback conditions. Second, although the ipsilateral precuneus in the MT condition had a higher beta value than in the normal visual feedback conditions, its 90% confidence interval contained zero, which meant no significant change from the baseline statistically. Therefore, the interpretation that MT induces the activation of the precuneus should be revisited. An increasing number of studies have revealed that the precuneus is activated in self‐referential tasks, especially in visual information processing such as mental imagery and mental navigation (Cavanna & Trimble, 2006). On the other hand, the precuneus is thought to be a part of the DMN and to be deactivated when engaging in goal‐directed and attention‐demanding tasks (Cavanna & Trimble, 2006; Raichle, 2015). Taken together, the activity of the DMN reflects a dynamic balance between the self‐referential status and attention effort (Raichle, 2015). MT requires participants to perform movements with one hand (unaffected hand for patients with stroke), and the incongruent visual feedback may result in some visual information processing, for example, illusionary motor imagery (Deconinck et al., 2015). In the study by Wang, Fritzsch, Bernarding, Holtze, et al. (2013), their results showed that MT did not change the activity of the precuneus, but normal visual feedback could deactivate the precuneus. Although Mehnert et al. (2013) found increased HbO by mirror visual feedback compared to baseline in the precuneus, the no visual feedback condition showed activation as well instead of deactivation which is not consistent with Wang, Fritzsch, Bernarding, Holtze, et al. (2013). However, in our study, we found that all conditions deactivated the precuneus and no significant difference was found among the conditions. We consider that this was due to the motor tasks we employed. In previous fMRI studies (Matthys et al., 2009; Wang, Fritzsch, Bernarding, Holtze, et al., 2013) and a fNIRS study (Mehnert et al., 2013), finger tapping was used, while we employed more complex motor tasks involving more finger joints and fine motor control. Therefore, we observed deactivation in all conditions, which might be due to the dominance of attention effort toward motor tasks and the effect of self‐referential visual information processing being weakened.

To conclude, our study showed that mirror visual feedback appears to induce higher activation than normal visual feedback in the ipsilateral SMC and SMA. The functional connectivity between the ipsilateral SMA and SMC is likely to be strengthened when receiving MT. In addition, mirror visual feedback seems to draw participants' attention toward the untrained side by activating the SPL. Compared with MMT, TMT showed a higher effect on activating the ipsilateral SPL due to the complexity of the motor task. We did not find any significant change of the precuneus induced by MT, suggesting that the precuneus may not be an essential component of the MT‐related neural network.

## AUTHOR CONTRIBUTIONS

Zhongfei Bai and Kenneth N. K. Fong involved in the conception and design of the study. Kenneth N. K. Fong performed the administrative support. Zhongfei Bai and Jiaqi Zhang collected the data. Zhongfei Bai and Zhishan Hu organized and performed the data analysis and interpretation. Zhongfei Bai and Kenneth N. K. Fong wrote the manuscript and finally approved the manuscript.

## Data Availability

The data that support the findings of this study are available on request from the corresponding author. The data are not publicly available due to privacy or ethical restrictions.
